# Supplementary material for: Novel Antimicrobial Peptide “Octoprohibitin” against Multidrug Resistant Acinetobacter baumannii
Source: Pharmaceuticals (Basel). 2022 Jul 27;15(8):928. doi: 10.3390/ph15080928 (PMC9415640; doi:10.3390/ph15080928)
Supplement: Supplementary file 1 [file pharmaceuticals-15-00928-s001.zip › pharmaceuticals-1785196-supplementary.pdf]

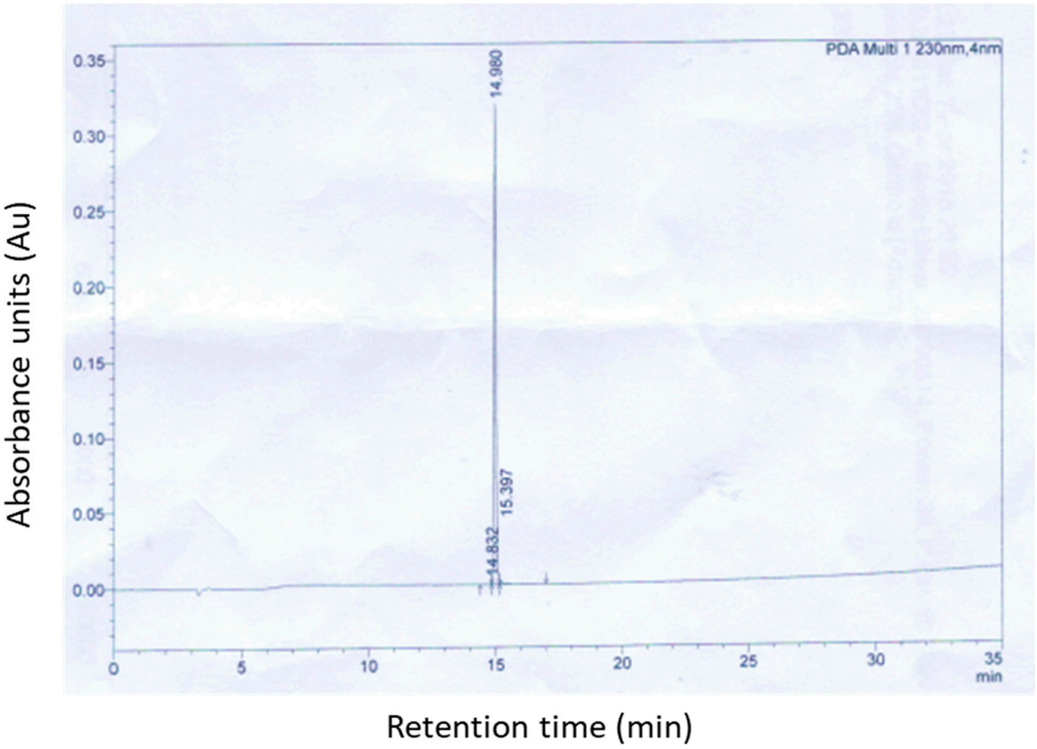


Figure S1. High-performance liquid chromatography (HPLC) mass spectrophotometry chromatographs of Octoprohibitin.

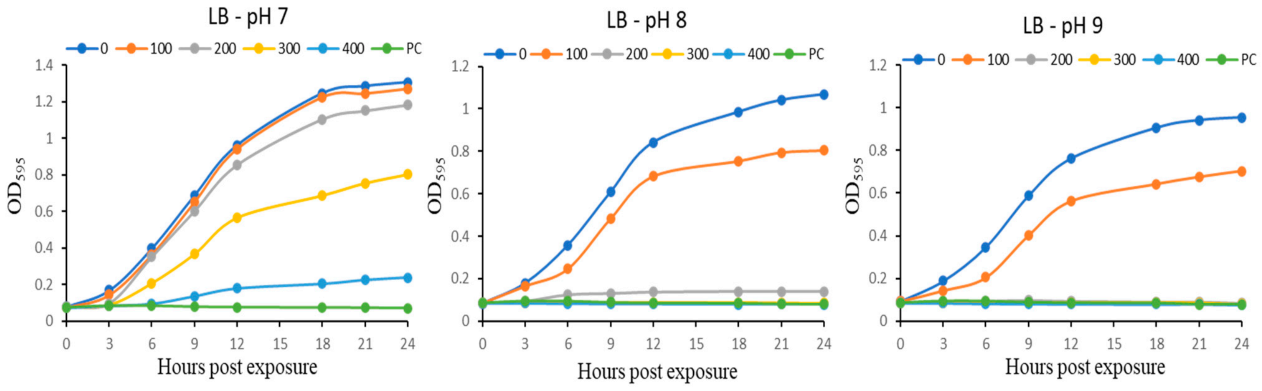


Figure S2. Time kill kinetic analysis of *A. baumannii* with Octoprohinitin (0 – 400 µg/mL) at pH 7, 8, and 9.
